# *Oenanthe millefolia* (Umbelliferae), a new species record for the Turkish and Greek Flora

**DOI:** 10.3897/phytokeys.70.9093

**Published:** 2016-09-20

**Authors:** Ebru Doğan Güner, Mehtap Tekşen, Barış Bani

**Affiliations:** 1 Health Services Vocational School, Gazi University, 06830, Ankara,Turkey; 2 Department of Biology, Faculty of Science and Arts, Aksaray University, 68100 Aksaray, Turkey; 3 Department of Biology, Faculty of Art and Sciences, Kastamonu University, 37150 Kastamonu, Turkey

**Keywords:** Anatomy, Balkan flora, Bulgaria, Greece, morphology, pollen, Oenanthe, taxonomy, Turkey

## Abstract

*Oenanthe millefolia* (Apiaceae), which is presented as a new recorded species for the Turkish flora, was discovered for the first time in Kırklareli province of Turkey. It is also reported as a new species for the Greek flora based on an unidentified specimen which was collected from the Thrace region of Greece. In this study, an expanded morphological description, the geographical distribution, the habitat properties and the ecological features of the species are exhibited with illustrative figures. Moreover, the micromorphological and anatomical characters of the fruits and the anatomical properties of the stem, petiole, leaves and the palynological features of *Oenanthe millefolia* are determined and described for the first time.

## Introduction

The genus *Oenanthe* L. is represented by nearly 35-40 species in the world and 8 species in Turkey (Hedge and Lamond 1972, Duman 2000). According to Menemen (2012), *Oenanthe incrassans* Bory & Chaub., which is considered as a synonym of *Oenanthe pimpinelloides* L. (Hedge and Lamond 1972), was uncertainly recorded from Turkey. However, the first author of this paper (Doğan Güner 2016) recently confirmed its occurrence in Turkey.

Some unusual specimens were collected from the Kırklareli province of Turkey during a project dealing with the revision of Turkish representatives of the genus *Oenanthe*. These specimens were clearly different from all the taxa of the genus distributed in Turkey (Hedge and Lamond 1972, Duman 2000). After consulting literature dealing with the flora of the adjacent regions and checking the herbarium vouchers, these materials were preliminarily identified as *Oenanthe millefolia* Janka (Cook 1981). However, these specimens were also similar to *Oenanthe bulgarica* Velen., which was described by Velenovsky (1898). Detailed investigations of literature and the herbarium vouchers show that *Oenanthe millefolia* and *Oenanthe bulgarica* are morphologically very similar taxa (Velenovsky 1898, Hedge and Lamond 1972, Cook 1981, Duman 2000). Moreover, *Oenanthe bulgarica* has already been synonymised under *Oenanthe millefolia* by Gandoger (1910) in his “Novus conspectus florae Europae”. With this species, the total number of the *Oenanthe* species in Turkey is now 10.

The aim of this study is to describe morphological, anatomical, palynological and fruit micromorphological properties of the species.

## Methods

The specimens, collected during the field studies in Igneada-Kirklareli region in 2014 and 2015, were checked with the related literature (Velenovsky 1898, Gandoger 1910, Hedge and Lamond 1972, Cook 1981, Duman 2000).

The specimens of *Oenanthe millefolia* were compared with the type specimen and the other representative vouchers kept in GOET, E, SOM, W, and WU herbaria (abbreviations following Thiers 2016).

All the samples which were used for the anatomical studies were fixed in 70% ethanol during the field works. They were stained using sartur reagent according to the method described by Çelebioğlu and Baytop (1949). The stained samples were examined under an Olympus E330 microscope and the anatomical properties were clarified. Moreover, related literature was used for the explanation of the anatomical characters (Mauseth 1988, Dickison 2000).

For the palynological studies, pollen slides were prepared according to the Wodehouse method (1935) and were examined under a light microscope (LM). Different 30 pollen grains were measured by using a Leica DM3000 microscope. The pollen samples and mericarps were directly placed on aluminium stubs and coated with gold with Polaron SC 502 Sputter Coater device and observed with the Jeol JSM 6490LV model scanning electron microscope (SEM). For the palynological and micromorphological terminology, Moore et al. (1991), Punt et al. (2007), Hesse et al. (2009) and Doğan Güner et al. (2011) were used.

## Results

### Oenanthe millefolia

Taxon classificationPlantaeApialesApiaceae

Janka Oesterr. Bot. Z. 22: 177-178, 1872

**Figure 1.**
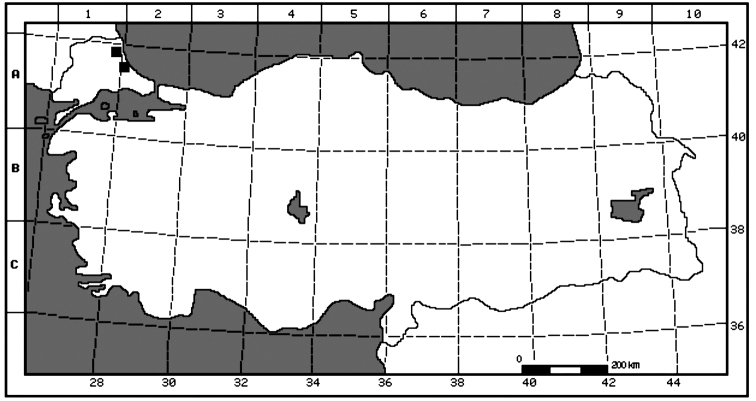
Distribution map of *Oenanthe millefolia* (■) in Turkey.

**Figure 2.**
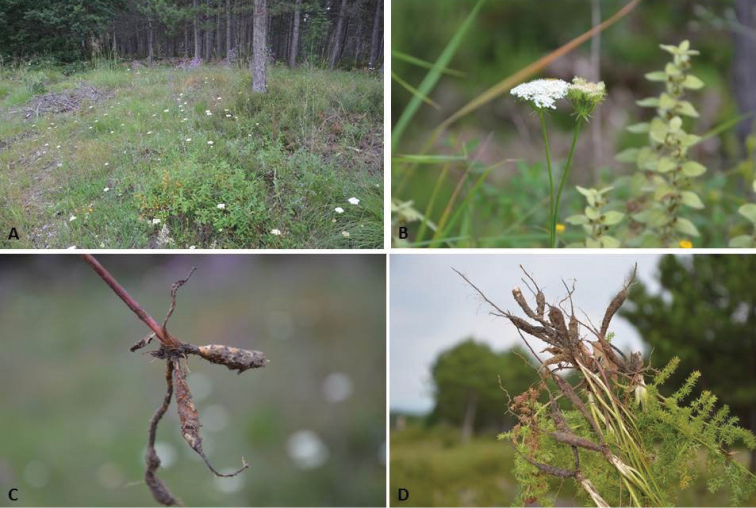
*Oenanthe millefolia* (E.Doğan Güner 2044) **A** habitat **B** inflorescens **C** root system **D** root system and basal leaves.

**Figure 3.**
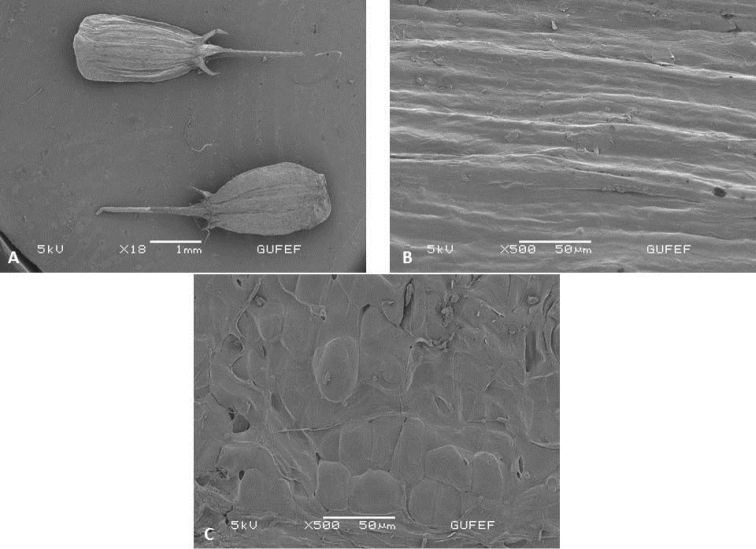
SEM micrographs of mericarp in *Oenanthe millefolia* (E. Doğan Güner 2101) **A** general view **B** dorsal view **C** ventral view.

**Figure 4.**
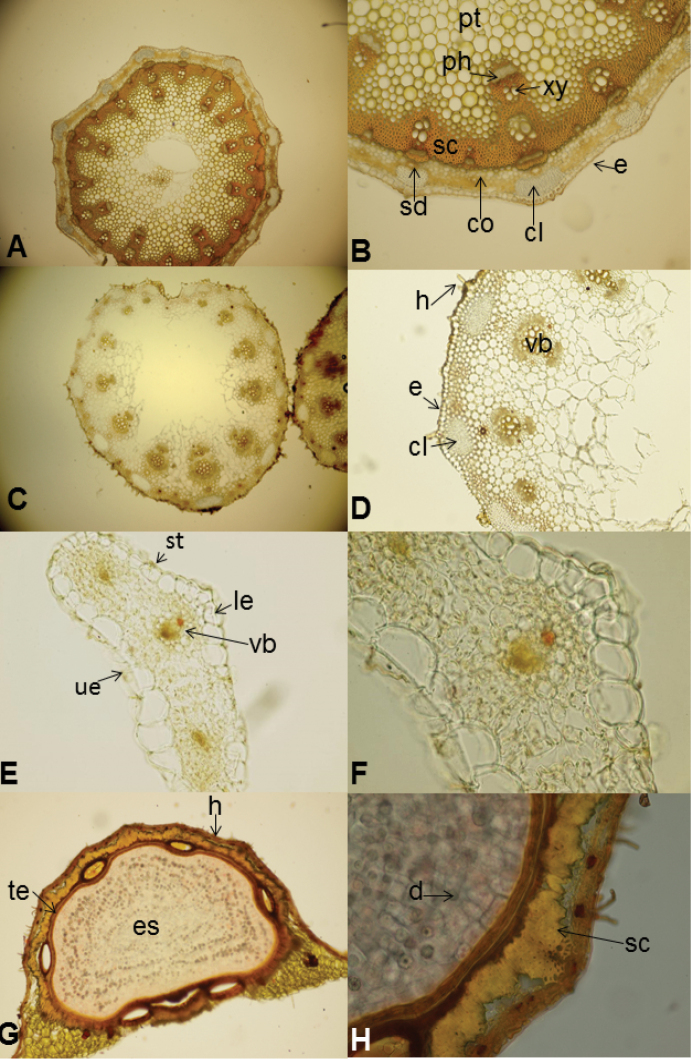
Anatomical structure of *Oenanthe millefolia* (E. Doğan Güner 2044) **A–B** Cross sections of stem (10 × 5), (10 × 20) **C–D** Cross sections of petiole (10 × 5), (10 × 20) **E–F** Cross sections of leaves (10 × 10), (10 × 40) **G–H** Cross sections of mericarp (10 × 10), (10 × 20). (Legend: cl: collenchyma, co: cortex, e: epidermis, es: endosperm, h: hair, le: lower epidermis, ph: phloem, pt: pith, sc: sclerenchyma, sd: secretory duct, st: stoma, t: testa, ue: upper epidermis, xy: xylem, v: vittae)

**Figure 5.**
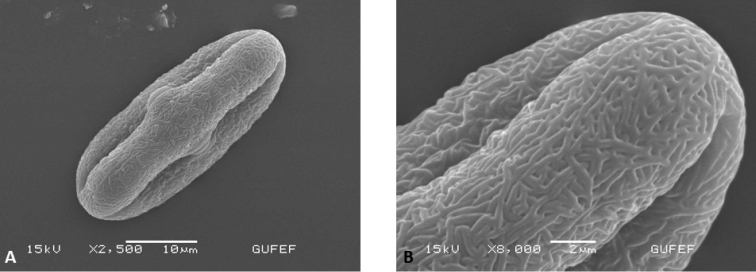
The pollen of *Oenanthe millefolia* (E. Doğan Güner 2044) **A** polar view **B** pollen grain with rugulate.

Figures 1 , 2 , 3 , 4 , 5

= Oenanthe bulgarica Velen. Fl. Bulg. Suppl. 1: 127, 1898.

#### Specimens examined.

**BULGARIA**. in pratis inter Kalofer et Karlova ad ped. austral. m. Balkan Thraciae, ubi specimina nondum bene efflorata legi d. 2. Junii 1871 (holotype GOET!); In graminosis pagum Susam. July 1910, V. Stribrny s.n. (W!) *(Oenanthe bulgarica)*; Kreis Sliven, Stara Planina (Balkan-Gebirge), Kotlenska Planina (Kotel-10 Str.-km S Kotel Richtung Gradec, Balkan), Rastplatz mit Brunnen, *Quercus*-Mischwald ca. 450 m s.m., 09 August 1978 Kalk., F. Ehrendorfer, F. Sorger, D. Fürnkranz, M.A. Fischer, A. Öztürk s.n. (WU!); Stara Zagora district, around *Quercus robur* forest, in Kilimite area, 26.06.1958, Ivan Ganchev (photo SOM!); South Black Sea coastline, oak forest between Ahtopol and Sinemorets, 09.05.2004, A. Petrova & B. Assyov (photo SOM!); Strandzha mountain, oak forest, by the roadside, Tsarevo-Malko Tarnovo, at fountain, west of Izgrev village, 09.05.2004, A. Petrova & B. Assyov (photo SOM!); Thracian Lowland, pasture, northeast of Malevo village, Haskovo district, 11.06.2004, A. Petrova (photo SOM!); Eastern Rhodopi, above Potocharka village, in forest of *Carpinus orientalis*, *Fraxinus ornus* and *Paliurus spina-christi*, 08.06.2006, D. Dimitrov (photo SOM!); Stara Zagora district, near Sarnevo village, in oak forest, 08.06.1960, Iv. Ganchev, St. Denchev (photo SOM!); St. Iliya hills, 22.07.1964, Iv. Ganchev, St. Denchev (photo SOM!).

**GREECE**. 3 km from Petrota along road to Pentalofos, open woodland of *Quercus frainetto*, 180 m, 41° 40´ N 26° 09´ E, 12 June 1991, Strid and Kit Tan 31802 (E!).

**TURKEY**. Kırklareli: Demirköy-Iğneada, to 5–10 km Iğneada, under *Pinus sylvestris* forest, 273 m, 18 July 2014, E. Doğan Güner 2044 & B. Bani (GAZI!); ibid., 03 August 2014, E. Doğan Güner 2075 & B. Bani (GAZI!); Kırklareli: Sarpdere–Armutveren, under *Pinus sylvestris* forest, 33 m, 19 June 2014, E. Doğan Güner 2046 & B. Bani (GAZI!); Tekirdağ: Saray, Kıyıköy district, under *Quercus* forest, 247 m, 16 June 2015, E. Doğan Güner 2101 & B. Bani (GAZI!).

#### Description.

Perennial, 40–70 cm tall, herb, with thickened, fusiform or oblong tubers, tubers generally at stem base, rarely far away. Stem erect, simple or 3 times branched above, hollow, furrowed, minutely scabrid below, glabrous above. Basal leaves lanceolate or oblong in outline, 2-3 pinnate, 17–45 × 5–9 cm, leaves lamina longer than petiole; segments of lamina opposite at rachis, deeply pinnatisect, ultimate segments linear or elliptic up to 6 × 1 mm, excurrent into a setaceaus tip. Upper leaves similar to basal one, but only a few which reduce upwards. Umbel with 12–18 slender rays of sub-equal length, up to 2 cm, becoming slightly thickened in fruit. Umbels 5 cm diam. at flowers and 3 cm diam. at fruit. Bracts 8-9, lanceolate, 8–10 × 1.5–2 mm. Umbellules conical with unequal thickened pedicels in fruit, 20-30 flowered, about 1–1.5 mm diam., pedicels of sterile flowers longer than fertile ones. Bracteoles 9–13, elliptic-linear, 2–3.5 × 0.5–1.5 mm. Petals radiating, white, cordate, to 2.5 mm long. Sepals ovate, 0.3–0.4 mm, acuminate at apex. Filaments at least two times longer than petals. Stylopodium is conical and not exceeding calyx teeth. Styles about as long as the body of the fruit (ca. 3 mm), erect. Fruit ovate to cylindrical, 3 × 2 mm, striate, laterally and base of fruit slightly spongiose margin.

#### Distribution, habitat and ecology.

*Oenanthe millefolia* is distributed in Bulgaria, North-eastern Greece and European Turkey (Fig. 1). No threat factor was observed against the habitat of the species. The populations are represented by many healthy individuals. The flowering time is between June and July, the fruiting time is August. It grows on clearings of *Pinus* and *Quercus* forest between the altitudes of 240–450 m and shares the same habitat with the species of *Oenanthe pimpinelloides* L., *Helianthemum racemosum* (L.) Pau, *Trachystemon orientalis* (L.) G. Don., *Crupina vulgaris* Cass, Pinus nigra Arn. subsp. pallasiana (Lamb.) Holmboe, *Pulicaria dysenterica* (L.) Bernh., and *Anthemis altissima* L.

#### Mericarp macromorphology and micromorphology.

Mericarps have three dorsal and two lateral primary ribs. The lateral ridges are more prominent and broader than the dorsal ones. The lateral ridges extend towards the base and cover the base of mericarp. Sepals are generally distinctive and persistent in *Oenanthe* species. Stylopodium is conical and not exceeding calyx teeth. Stylopodium ending with style is almost as long as the fruit. The surface ornamentation of the pericarp is longitudinally striate. The pattern is formed by rectangular cells. Stomatal cavities are observed on the pericarp surface and the density increases towards the calyx (Fig. 3). The style surface is ribbed.

#### Anatomy.

*Stem anatomy*: Stems are circular and slightly 7–8 ribbed in cross sections. There is a thin layer of cuticle on the top surface and a single-line epidermis composed of rectangular cells underneath. Collenchyma cells are grouped at the edges. Cortex parenchyma cells are located around disordered collenchyma cells. Collenchyma and parenchyma cells are identified as two layers between edges. Collenchyma has 4–5 rowed, small, and circular cells. Parenchyma has 1–2 rowed, large and circular cells. Secondary slight edges are located between the edges with collenchyma cells. Secretion canals exist in cortex under the collenchyma layer. 5–6 cells in one line surround the canals. Endoderm cell walls are slightly thick one-line oval cells. Vascular bundles are embedded between the cortex and the pith. There are 6–7 rows of sclerenchymatic cell layers between the vascular bundles. Peripheral vascular bundles which are collateral, are large against the edges and small in between the edges. Central vascular bundles are connected with peripherals by sclerenchymatic tissue. Secretion canals are also found under vascular bundles. Slight thickening is seen in pith parenchyma cells. Pith cells have various sizes with large inter-cellular spaces (Fig. 4A–B).

*Petiole anatomy*: Petiole is almost straight in cross section, ovoid in outline and slightly-canaliculate on the lower surface. Scabrid hairs rarely occur between cubic epidermis cells. Cuticle, epidermis and collenchyma structures are designed in almost the same manner as the stem. Secretion canals between collenchyma and concentric vascular bundles are significant. Xylem elements are dominantly distributed. The pith is composed of large circular cells with a hollow centre (Fig. 4C–D).

*Leaf anatomy*: The epidermal layer consists of rectangular or circular cells in both adaxial and abaxial directions. Stoma exist on both surfaces. There are large respiratory spaces under the stomata. Mesophyll is composed of two–row palisade and one or two–row sponge parenchyma cells. Large ventilation spaces exist between sponge parenchyma cells. Xylem elements are located through the abaxial side and phloem elements are located through the adaxial side (Fig. 4E–F).

*Fruit anatomy*: The fruit is schizocarp with two mericarps. The pericarp forms a thin layer around the endocarp and seed. There are single-line and horizontally located epidermal cells on the surface. The mesocarp is formed by 2–3–row small cells. Both epidermis and mesocarp cells have significant thickness. Five ridges are seen on each mericarp. Vascular bundles are located on these ridges. They are reduced. Secretion canals are located on vascular bundles. Pericarp is surrounded with sclerenchymatic tissue which makes a continuous ring up to the carpophore. The sclerenchyma layer is composed of irregular cells with thick walls. There are 4 dorsally and 2 ventrally vittae. Oval-shaped vittae are located in the vallecular region. Endoderm is located as one line under the vittae and seems to be integrated with the testa. The seed is composed of endosperm and testa with a thickened cell wall. Endosperm contains large quantities of lipid and protein. There are many druse crystals in the endosperm. When the section is taken from the middle of the mericarp, the embryo cannot be observed because it is small and close to the tip (Fig. 4G–H).

#### Pollen morphology.

The pollen grains are isopolar symmetric, the aperture is tricolporate type. The pollen shape is prolate with an elliptic equatorial outline, polar axis 29.5–33.5 µm, equatorial axis 15–18 µm. The ornamentation is rugulate. The colpus length is 18–27 µm and width is 0.5–2 µm. The pore length is 4–6 µm and width is 4–6 µm. The exine subtectate is 0.75–1 µm (on equator and polar), the intin is 0.75–1.25 µm (on equator and polar) (Fig. 5).

## Discussions

After detailed investigations on the descriptions of *Oenanthe millefolia* and *Oenanthe bulgarica*, it was found that these two taxa are conspecific. Gandoger (1910) also previously recognised *Oenanthe bulgarica* as the synonym of *Oenanthe millefolia*. However, Cook (1981) did not mention the synonymy of *Oenanthe bulgarica* under *Oenanthe millefolia* in Flora Europaea.

The leaf lamina of *Oenanthe millefolia* is deeply pinnatisect, comprising primer segments with shorter and denser ultimate segments. The primer segment is also remotely and evenly distributed to the apex (Fig. 2D). This leaf morphology clearly resembles the leaf of *Oenanthe tricholoba* Greuter (Greuter 2012). *Oenanthe tricholoba* has however ± globular root tubers which are distant from the base of stem (not fusiform or oblong tubers and not very close to the base of the stem).

In this study, the morphological description of the species has been expanded with investigated specimens (especially our collections, the specimens of W and WU herbaria and the photographs of the specimens from GOET, SOM, E herbaria). Some of the morphological characters show high variations. In our collected specimens, root tubers are generally close to the base of the stem but some of them are remote (not only at base of stem), basal leaves 2–3 pinnate (not 2–pinnate) and umbels with 12–18 rayed (not 5–15 or 10–16).

During the studies on the specimens of different herbaria, we realised that one unidentified specimen deposited at E herbarium is identical to *Oenanthe millefolia*. This specimen was collected from Greece. With this additional record, the distribution of *Oenanthe millefolia*, so far known as a Bulgarian endemic, has been extended southwards to Turkey and Greece. Therefore, the species should be considered as a Balkan endemic.

The species of *Oenanthe* in Turkey, mostly prefer wetlands and humid areas. It has however been observed that *Oenanthe pimpinelloides* is distributed in dry areas such as under trees or open woodlands in addition to the aquatic habitats. Moreover, *Oenanthe millefolia* is observed only in dry areas with *Oenanthe pimpinelloides*.

This study comprises the discovery of *Oenanthe millefolia* in the Thracian regions of Turkey and Greece. To date, there has been no comprehensive study dealing with *Oenanthe millefolia*. This paper provides micromorphological, anatomical and palynological features of the species along with its expanded morphological description. The findings of this study can contribute to further taxonomical investigations on both the genus *Oenanthe* and family Apiaceae.

## Supplementary Material

XML Treatment for Oenanthe millefolia
